# Characterization of three alternative transcripts of the *BRCA1* gene in patients with breast cancer and a family history of breast and/or ovarian cancer who tested negative for pathogenic mutations

**DOI:** 10.3892/ijmm.2015.2103

**Published:** 2015-02-16

**Authors:** GAETANA GAMBINO, MARIELLA TANCREDI, ELISABETTA FALASCHI, PAOLO ARETINI, MARIA ADELAIDE CALIGO

**Affiliations:** 1 Department of Translational Research and New Technologies in Medicine, University of Pisa, Pisa 56126, Italy; 2 Section of Genetic Oncology, Santa Chiara University Hospital, Pisa 56126, Italy; 3 Fondazione Pisana per la Scienza - ONLUS, Pisa 56126, Italy

**Keywords:** *BRCA1*, *BRCA2*, aberrant splicing, naturally occurring, alternative transcript

## Abstract

The study of *BRCA1* and *BRCA2* genes and their alterations has been essential to the understanding of the development of familial breast and ovarian cancers. Many of the variants identified have an unknown pathogenic significance. These include variants which determine alternative mRNA splicing, identified in the intronic regions and those are capable of destroying the splicing ability. The aim of this study was to detect BRCA1/BRCA2 aberrant transcripts resulting from alternative splicing, in women with a known family history and/or early onset of breast and/or ovarian cancer, tested wild-type for BRCA1 and BRCA2. The identification and characterization of aberrant transcripts through the analysis of mRNA levels in blood lymphocytes may help us to recognize families otherwise misclassified as wild-type *BRCA1* and *BRCA2*. Blood samples were collected from 13 women that had a family history of breast and/or ovarian cancer and tested negative for pathogenic mutations in the *BRCA1* and *BRCA2* genes. Total RNA was analyzed for the presence of BRCA1 and BRCA2 naturally occuring and pathological transcripts using RT-PCR. In 2 out of the 13 samples, 2 alternative transcripts of the *BRCA1* gene were identified. These were probably pathogenic as they lacked exon 17 and exon 15, respectively, giving rise to a truncated protein. In addition to these, we identified the Δ17–19 transcript in 1 patient, which gives rise to a protein with an in-frame deletion of 69 amino acids. In conclusion, this study on alternative transcripts of the *BRCA1* and *BRCA2* genes revealed the presence of isoforms (prevalence of 15%) in blood samples from women with breast and ovarian cancer that were probably pathogenic, that were not detected by conventional methods of mutation screening based on direct sequencing of all coding regions, intron-exons junctions and MLPA analysis.

## Introduction

Inactivating mutations in the *BRCA1* (MIM 113705) and *BRCA2* (MIM 600185) genes confer a high risk of developing breast and ovarian cancer (1,2). Both genes have a contribution of approximately 16% to the risk of familial breast cancer (3). Genetic testing for *BRCA1* and *BRCA2* provides valuable information for determining the clinical management of patients with breast/ovarian cancer. However, the data provided are difficult to interpret due to the identification of many DNA variants of unknown pathological significance or unclassified variants (UVs) that hamper genetic counseling in hereditary breast and ovarian cancer (HBOC) (4).

UVs have the potential to alter protein function by altering the coding sequence of a transcript, or the level of the gene transcript by disrupting regulatory regions in promoters, untranslated regions, exons or introns (5). Such regulatory variants include those affecting the normal splicing of *BRCA1* and *BRCA2*, many of which have been shown to be clinically significant using cDNA studies and multifactorial likelihood analysis methods that combine bioinformatics, as well as pathological and clinical information (6,7).

Assessing the impact of UVs on splicing is key to determining their pathogenicity. The accuracy of pre-mRNA splicing is determined by the recognition of well known 5′ and 3′ splice site consensus sequences. However, more discrete elements are also involved, such as exonic splicing enhancers (ESEs) that enhance pre-mRNA splicing when present in exons (8). As a result, each UV may potentially affect normal pre-mRNA splicing and be deleterious through the disruption of consensus sequences, or through the creation of *de novo* sequences or the alteration of splicing regulatory elements (9). Several *BRCA1* isoforms have been identified in different tissues; however, their functional significance is not yet fully understood (10–15).

A recent study on the comprehensive annotation of BRCA1 splice junctions identified 63 independent alternative splicing events in RNA samples from healthy control individuals. Among these, 10 were predominant (Δ1Aq, Δ5, Δ5q, Δ8p, Δ9, Δ9–10, Δ9-10-11, Δ11q, Δ13p and Δ14p) and represented 5–30% of the full-length signal; 48 were minor and 5 were non-classifiable events (16).

Alternative splicing is a highly coordinated process. Mutations destroying the 5′ and 3′ splice site consensus sequences may alter the splicing patterns of one or more transcripts, interrupting the production or function of the encoded protein (17). A large number of pathological transcripts of the *BRCA1* and *BRCA2* genes, deriving from a mutation in the consensus regions, have been described in the literature (18).

The aim of this study was to identify aberrant transcript variants resulting from the alternative splicing of *BRCA1* and *BRCA2* genes in RNA extracted from blood lymphocytes from women with a family history of and/or early onset breast and/or ovarian cancer, in which genomic pathogenic alterations in *BRCA1* and *BRCA2* have not been detected by conventional analysis.

The analysis of all possible transcripts of the *BRCA1/BRCA2* genes may allow us to uncover mRNA splicing defaults overlooked by conventional protocols and to confirm that the alternative splicing of the *BRCA1* and *BRCA2* genes plays an important role in *BRCA1/2*-driven tumorigenesis. This approach has the potential to complete the process of the characterization of mutations of *BRCA1* and *BRCA2* in HBOC.

## Materials and methods

### Sample acquisition

A total of 13 blood samples were collected from women with a family history of and/or early-onset breast and/or ovarian cancer and who tested negative for pathogenic mutations in the *BRCA1* and *BRCA2* genes. Sample data details and characteristics are presented in Table I.

The BRCAPRO (http://www4.utsouthwestern.edu/breast-health/cagene/) and BOADICEA (https://pluto.srl.cam.ac.uk/cgi-bin/bd2/v2/bd.cgi) (19,20) programs were used, calculating *a priori* mutation carrier risk of >10% for each patient.

Ten blood samples from healthy women, aged 25–45 years, with no family history of breast and/or ovarian cancer, were used as the controls. All patients and healthy donors were recruited at the Interdepartmental Centre for Cancer Genetics, University Hospital of Santa Chiara in Pisa, Italy between 2004 and 2011. Prior to enrollment, informed consent for genetic analysis was obtained from all patients. All the experiments carried out complied with the current laws of the country in which they were performed (Italy).

### RNA extraction and cDNA synthesis

All samples were subjected to total RNA extraction in 2 steps: peripheral blood mononuclear cells (PBMCs) were isolated from ethylenediaminetetraacetic acid (EDTA)-treated peripheral blood by standard density gradient centrifugation (Ficoll 15%; Bio-Rad Medical Diagnostics GmbH, Dreieich, Germany) and RNA extraction was obtained using a TRI Reagent kit (Molecular Research Center, Inc., Cincinnati, OH, USA) as recommended by the manufacturer. Isolated RNA was quantified using a NanoDrop spectrophotometer (NanoDrop Technologies, Inc., Wilmington, DE, USA) and examined for integrity on a 1.5% agarose/formaldehyde gel containing 0.5 *μ*g/ml ethidium bromide (RNA Analysis Notebook; Promega Corp., Madison, WI, USA). First-strand cDNA was synthesized from at least 1,000 ng of total RNA using an oligo(dT) primer or random primer and SuperScript III reverse transcriptase (Invitrogen, Carlsbad, CA, USA) according to the manufacturer’s instructions.

### Reverse transcription-polymerase chain reaction (RT-PCR)

**Figure 1:**
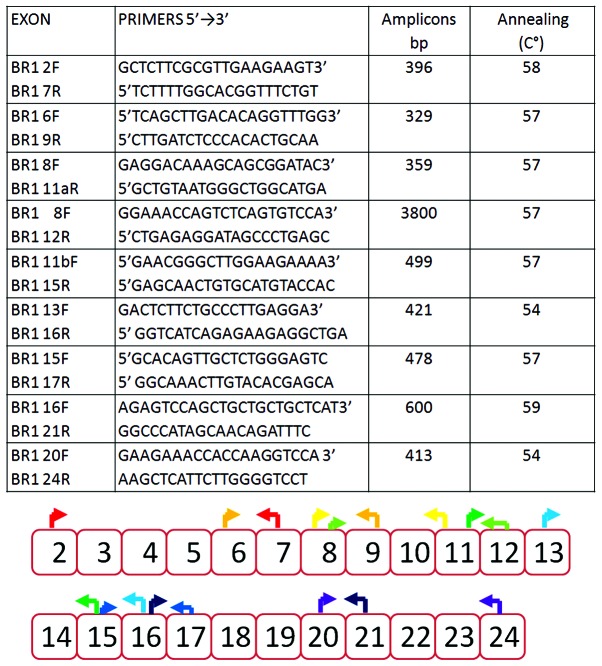
Primers used for the scanning of exons in the *BRCA1* gene. Primer pairs were used to amplify the cDNA covering the entire coding region of the *BRCA1* gene overlapping the exonic region.

**Figure 2:**
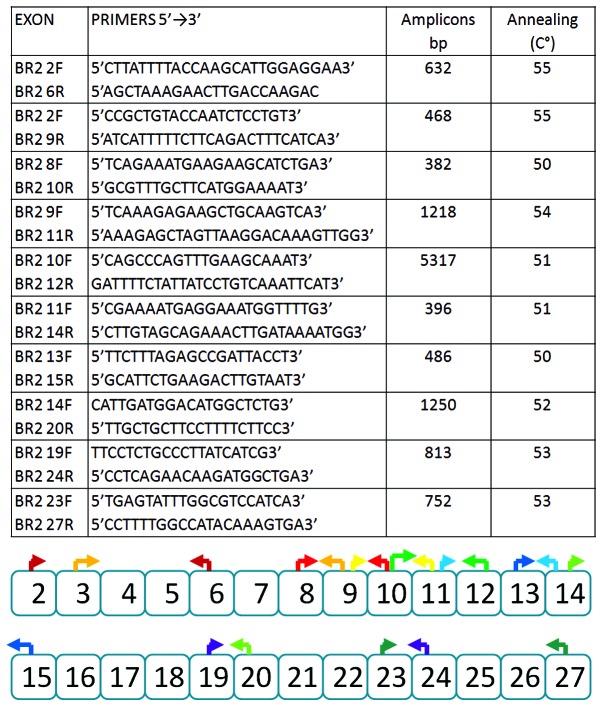
Primers used for the scanning of exons in the *BRCA2* gene. Primer pairs were used to amplify the cDNA covering the entire coding region coding of the *BRCA2* gene overlapping the exonic region.

To perform the amplification of naturally occurring transcripts of the *BRCA1* and *BRCA2* genes, we used multiple combinations of forward and reverse primer pairs to amplify overlapping regions of the mRNA and to cover the entire open reading frame (Figs. 1 and 2). For each PCR reaction, 2 *μ*l of cDNA were used. The PCR conditions were as follows: 5 min at 95°C followed by 4–45 cycles at 95°C for 1 min, melting temperature according to primer pair for 30 sec, 72°C for 1 min followed by 72°C for 1 min. To encompass multiple exons for large regions, Long Range PCR (Expand Long Template PCR system; Roche, Indianapolis, IN, USA) was used. The PCR products, eventually isolated on agarose gels, were sequenced on both strands using the BigDye Terminator v3.1 Cycle Sequencing kit and the 3130×l Genetic Analyzer (both from Life Technologies, Foster City, CA, USA). The electropherograms were analyzed using SeqScape Software v2.6 (Life Technologies).

### Multiplex ligation-dependent probe amplification (MLPA)

To exclude large genomic deletions in the *BRCA1* and *BRCA2* genes, MLPA (MRC-Holland, Amsterdam, The Netherlands) was performed as recommended by the manufacturer’s instructions. The electropherograms were analyzed using GeneScan (Life Technologies) and Coffalyser software (MRC-Holland).

## Results

The aim of this study was to identify alternative transcripts of the *BRCA1* and *BRCA2* genes resulting from aberrant splicing events. The analysis was conducted on 13 total RNA samples extracted from PBMCs from women with a family history of and/or early-onset breast and/or ovarian cancer, more specifically, 5 cases of hereditary breast cancer (HBC), 4 cases of HBOC, 1 case of bilateral carcinoma, 1 case of breast/ovarian cancer and 2 cases of early-onset breast cancer (Table I.)

All women were affected by breast cancer, 1 women by breast and ovarian cancer, 3 by bilateral breast cancer and 2 by early-onset breast cancer. A total of 10 control RNA samples from healthy women, aged between 25 and 45 years with no family history of any form of cancer, were also included in this study.

The genomic DNA of each patient was analyzed by direct sequencing of the entire open reading frame, 5′ and 3′UTRs and exon/intron junctions of both genes. Approximately 100 bps from the 5′ and 3′ end of each intron were sequenced. All cases tested negative for the presence of germline mutations in the *BRCA1* and *BRCA2* genes. The presence of variants of unknown pathological significance was also excluded from our analysis. The presence of large genomic deletions or rearrangements was excluded by MLPA of DNA extracted from the peripheral blood lymphocytes of all patients and the controls.

The *BRCA1* and *BRCA2* mRNA in each patient was analyzed by dividing the cDNA into 9 amplicons for *BRCA1* and 10 amplicons for *BRCA2*. The size of the full-length transcripts of the *BRCA1* (5,592 bp) and *BRCA2* (10,987 bp) genes prevents the analysis of the cDNA as a single amplicon.

### Naturally occurring transcripts of BRCA1

The cDNA of the *BRCA1* gene was amplified with primers localized in exonic sequences so that partially overlapping amplified products were obtained. The primers were selected in order to highlight all predominant naturally occurring alternative splicing isoforms and 28 of the minor transcripts, starting from exon 2, as previously reported by Colombo *et al* (16).

Under our experimental conditions, the following naturally occurring alternative transcripts were observed: the Δ9–10 transcript was detected in 3 samples, the Δ9 transcript was detected in 1 patient and Δ14p was detected in all the samples. Using the long range PCR approach, the following transcripts were detected: Δ11q (transcript lacking exon 11 except the initial 121 bp) in 10 samples and Δ9-10-11q (transcript lacking exons 9, 10 and 11 except the initial 121 bp) in 2 cases only.

### Naturally occurring transcripts of BRCA2

The cDNA of the *BRCA2* gene was amplified with primers localized in exonic sequences so that partially overlapping amplified products were obtained. A total of 5 predominant naturally occurring transcripts have been previously identified: Δ4, Δ4–7, Δ17–18, Δ18 and Δ20 (7,18,21). Under our experimental conditions, all 5 alternative transcripts were detected: More specifically, the Δ4 transcript was detected in 2 patients, the Δ4–7 transcript was detected in 2 patients, the Δ17–18 transcript was detected in 2 patients and the Δ18 transcript was detected in 7 patients. The Δ20 transcript was also detected in 1 case. All these BRCA1 and BRCA2 naturally occurring transcripts were detected in the controls.

### Abberant transcripts

In addition to the above-mentioned predominant naturally occurring transcripts, we detected 3 aberrant transcripts in the *BRCA1* gene in 2 patients (2 in patient P6 and 1 in patient P7). No aberrant transcripts were detected in the *BRCA2* gene.

**Figure 3:**
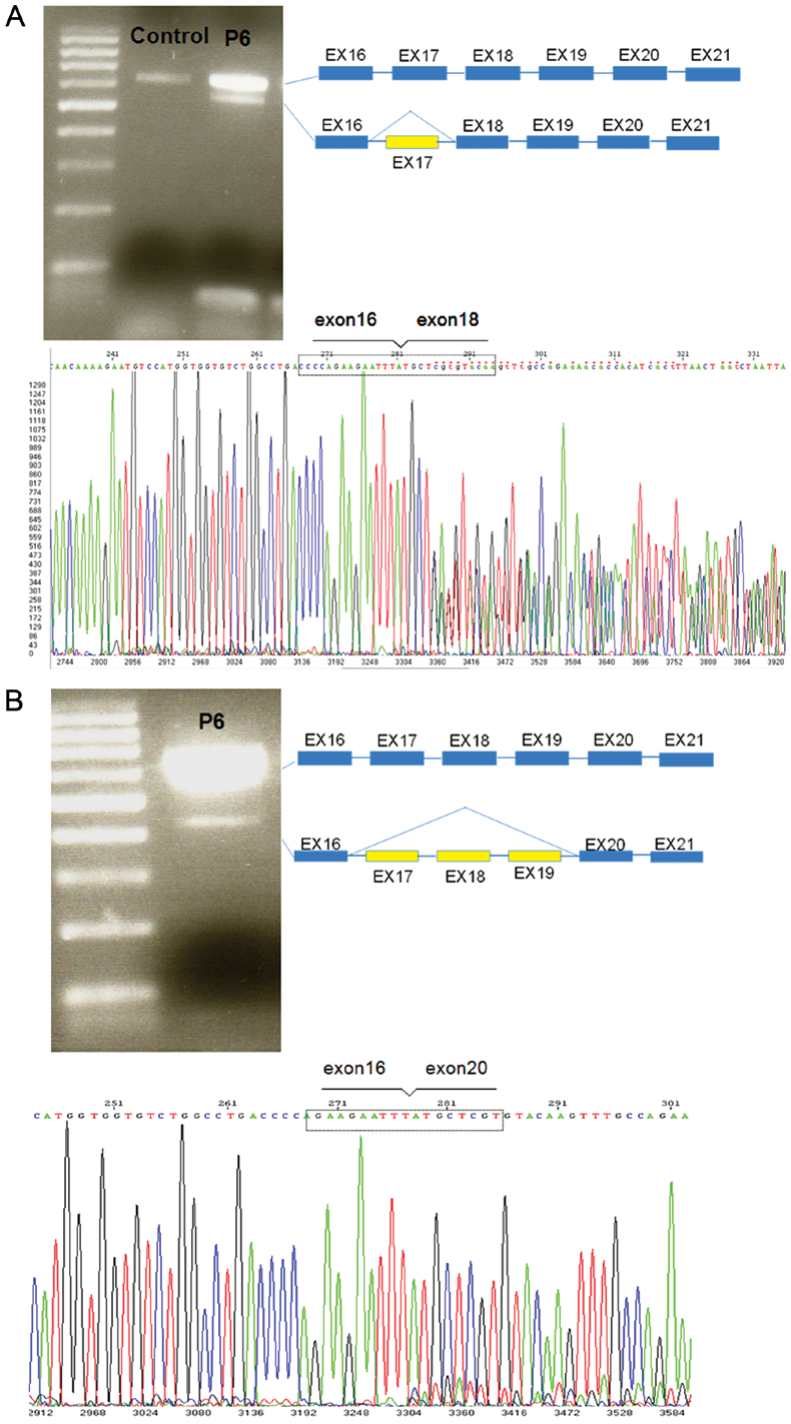
Alternative transcript isoforms in patient P6. Following the amplification of the cDNA of *BRCA1* with primers localized in exons 16 21, the wild-type (wt) transcript (~600 bp) and 2 additional bands of (A) ~500 bp and (B) ~400 bp were obtained. Sequencing analysis allowed us to detect the presence of a transcript lacking (A) exon 17 and another transcript lacking (B) exons 17, 18 and 19.

In patient P6, following the amplification of the cDNA of *BRCA1* with primers localized in exons 16 and 21 in addition to the full-length transcript (600 bp), 2 additional transcripts of approximately 500 and 400 bp were obtained. These transcripts were not detected in the 10 healthy control samples. The aberrant transcripts were gel-purified and then sequenced as described in the Materials and methods. Sequence analysis allowed us to detect the presence of a transcript lacking exon 17 and another transcript lacking exons 17, 18 and 19 (Fig. 3). The transcript containing the deletion of exon 17 produced an abnormal stop signal at codon 1673 (HGVS codification: p.Val1665Serfs*8) and then a truncated protein lacking the last 192 amino acids. The transcript containing the deletion of exons 17, 18 and 19 did not produce an abnormal stop signal, lost 207 nucleotides and retained the open reading frame, producing a protein lacking 69 amino acids.

**Figure 4:**
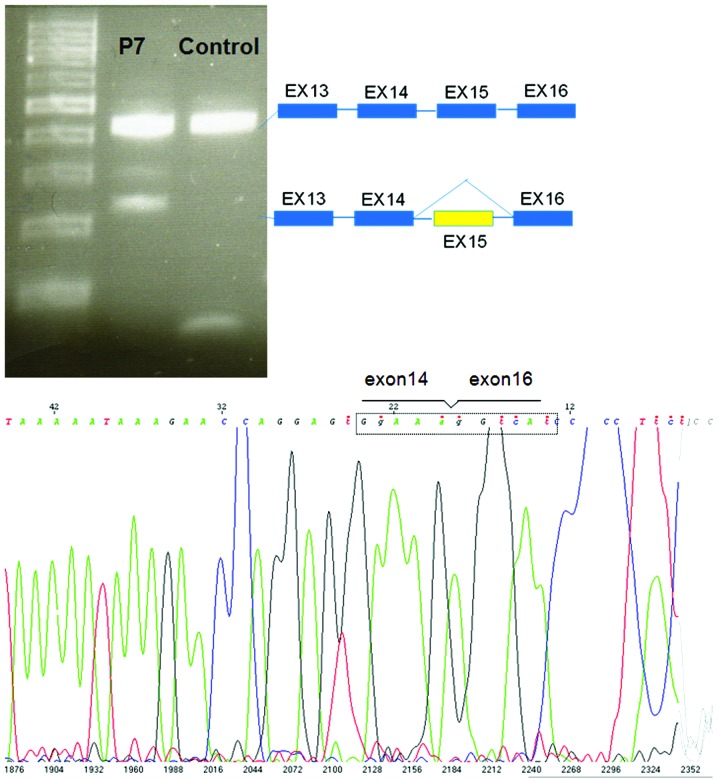
Alternative transcript isoform in patient P7. Following the amplification of the cDNA of *BRCA1* with primers localized in exons 13 and 16 with primers 13F and 16R, an additional band of ~420 bp was obtained. The fragment had a deletion of exon 15 as detected by direct sequencing.

In patient P7, following the amplification of the cDNA of the *BRCA1* gene with primers localized in exon 13 and 16 in addition to the full-length transcript of approximately 420 bp, an additional transcript of approximately 220 bp was obtained (Fig. 4). Direct sequencing of this transcript allowed us to detect an exon 15 deletion. The transcript containing the deletion of exon 15 produced an abnormal stop signal at codon 1510 (HGVS codification: p.Ser1496Glyfs*14) and then a truncated protein lackcing the last 405 amino acids.

### Phenotype-genotype correlation

Patient P6, at the age of 35 years, developed an infiltrating ductal carcinoma of the right breast, which was estrogen receptor-positive, progesterone receptor-negative and Her2/neu 3+, with loco-regional lymph node metastasis. The patient’s sister had been diagnosed with breast cancer of a similar phenotype at the age of 34. These were not the only cases of breast cancer in the family; the grandmother and maternal aunt (their mother’s twin sister) had also been afflicted by the disease. Segregation analysis of the aberrant transcript in this family was not possible as DNA samples were not available.

Patient P7 was diagnosed with breast cancer at the age of 55 years. The tumor was an infiltrating breast carcinoma of the right breast, and was estrogen receptor-positive, progesterone receptor-negative and Her2/neu-negative, without any lymph node metastasis. The patient’s mother had been affected by ovarian cancer at 77 years of age and 3 cases of breast cancer were reported in the family: 2 sisters of the mother and the mother’s cousin (36 years of age). On the mother’s side, an uncle had been affected by malignant melanoma, and an aunt by a brain tumor. These relatives were not available for co-segregation analysis, but this aberrant transcript was not detected in the 42-year-old healthy daughter.

## Discussion

This study focused on the possibility that a proportion of patients with HBOC have mutations in the *BRCA1* or *BRCA2* genes that affect splicing, but are not detectable through sequencing of the gene exons or intronic sequences near the intron-exon boundaries. Several studies have demonstrated that, due to variations in splice sites, *BRCA1/BRCA2* may generate truncated, non-functional proteins that may be associated with a predisposition to breast and ovarian cancer (22). However, to the best of our knowledge, no studies have evaluated the presence of *BRCA1/BRCA2* pathological transcripts in patients without mutations identified in the canonical splice sites or regulatory sequences.

Recently, a systematic description of ‘naturally occurring’ alternative splicing at the BRCA1 locus was conducted by the Evidence-based Network for the Interpretation of Germline Mutant Alleles (ENIGMA) consortium (16). This led to the annotation of 63 splicing events, of which 35 were novel findings, even though most of them are rather minor, and it is likely that some do not qualify as ‘naturally occurring’ events, suggesting that the characterization of the full complexity of *BRCA1* splicing requires further investigation (16).

The present study was performed in accordance with a standard assay design and detection methods formulated by ENIGMA Consortium members (23) in order to detect the predominant naturally occurring alternative splicing isoforms of *BRCA1*, as reported by Orban *et al* (13,14), which were the only data available at the moment of the study design. There is no standard assay design for the detection of the predominant naturally occurring alternative splicing isoforms of *BRCA2*; thus we referred to the transcripts described in Ensembl (http://www.ensembl.org/index.html).

Using this strategy, we detected the Δ9, Δ9–10, Δ11q, Δ9-10-11q and Δ14p *BRCA1* isoforms and the Δ4, Δ4–7, Δ17–18, Δ18 and Δ20 *BRCA2* isoforms. In addition these predominant *BRCA1* transcripts, we detected the Δ17 and the Δ17–19 isoforms in patient P6 and the Δ15 isoform in patient P7. No minor transcripts were detected for *BRCA2*.

The Δ17 transcript lost the open reading frame, leading to the formation of a truncated protein. The Δ17 transcript was not detected in any of the 10 healthy controls in our study; however, it has previously been found in 28 out of 103 (27%) independent control samples or technical replicas in the ENIGMA Consortium study (16). The Δ17 transcript, due to the genomic deletion of exon 17, has frequently been detected in Italian families (24) possibly as a result of a non-homologous rearrangement among ‘Alu repeats’. The Δ17 transcript is not derived from a genomic rearrangement as demonstrated by MLPA of the patient’s DNA. The Δ17 transcript, even if not derived from a genomic rearrangement or from aberrant mRNA maturation due to canonical splice site mutations, it has to be considered as pathogenetic as it encodes for a truncated protein.

The Δ17–19 transcript gave rise to a protein with an in-frame deletion of 69 amino acids. The Δ17–19 isoform was not detected in either our 10 healthy control samples or the 223 independent control samples and technical replicas in the ENIGMA Consortium study (16). Although the Δ17–19 transcript had an in-frame deletion, a recent study demonstrated that the expression of the human *BRCA1* alternative splicing variant, Δ17–19, in the MCF-7 cells resulted in an impaired assembly of DNA repair complexes and aberrant DNA damage response (25), thus contributing to an increased risk of developing cancer in the carrier subjects.

In patient P7, the *BRCA1* alternative transcript Δ15 was detected. The alternative Δ15 transcript lost the open reading frame and produced an abnormal stop signal to position p.Ser1510Glyfs*14, leading to a truncated protein lacking the last 405 amino acids. To evaluate the presence of Δ15 at the genomic level, we performed MLPA, which did not reveal any deletion. This isoform was not detected in our 10 healthy PBMC control samples or in the 8 PBMC samples analyzed by Colombo *et al* (16). They found this transcript in only 4 of 10 replicas derived from samples of leukocytes, PHA-stimulated peripheral blood leukocytes and lymphoblastoid cell lines (they did not specify if these were independent samples or replicas of the same sample).

In conclusion, the present study on 13 patients with a family history of breast and/or ovarian cancer detected 3 alternative transcripts, probably pathogenic and not predictable by genomic screening, in 2 patients. A number of studies have described these 3 transcripts as consequences of large genomic rearrangements or mutations in canonical splice sites (24–28). These aberrant alternative transcripts may undergo nonsense-mediated decay (NMD) as they give rise to truncated proteins (29); therefore, the accurate quantification of these alternative transcripts is crucial to discriminating what is considered pathological and what is neutral for clinical relevance. The relative abundance of the minor transcripts detected in this study may prove to be useful in evaluating their possible role in cancer predisposition and risk modification.

## Figures and Tables

**Table I:** Summary of patient data and family history of cancer.

Patient	Personal history (age at onset, years)	Family history		
		No. of breast cancer cases	No. of ovarian cancer cases	No. of cases of other types of cancer
P1	Br (57)	4	–	1
P2	Br (34)	1	–	1
P3	Br (43)	8	1	5
P4	Br (37)	1	1	4
P5	Br (55), Ov (61)	–	–	2
P6	Br (35)	3	–	1
P7	Br (55)	3	1	4
P8	Br bil (43)	4	1	3
P9	Br (56)	3	–	2
P10	Br bil (45 and 50)	4	–	9
P11	Br (25)	–	–	1
P12	Br (32)	–	–	1
P13	Br bil (38 and 48)	–	–	6

Br, breast cancer; Ov, ovarian cancer; bil, bilateral.

## References

[1] Miki Y, Swensen J, Shattuck-Eidens D, Futreal PA, Harshman K, Tavtigian S, Liu Q, Cochran C, Bennett LM, Ding W (1994). A strong candidate for the breast and ovarian cancer susceptibility gene BRCA1. Science.

[2] Wooster R, Bignell G, Lancaster J, Swift S, Seal S, Mangion J, Collins N, Gregory S, Gumbs C, Micklem G (1995). Identification of the breast-cancer susceptibility gene BRCA2. Nature.

[3] Stratton MR, Rahman N (2008). The emerging landscape of breast cancer susceptibility. Nat Genet.

[4] Sanz DJ, Acedo A, Infante M, Durán M, Pérez-Cabornero L, Esteban-Cardeñosa E, Lastra E, Pagani F, Miner C, Velasco EA (2010). A high proportion of DNA variants of BRCA1 and BRCA2 is associated with aberrant splicing in breast/ovarian cancer patients. Clin Cancer Res.

[5] Chatterjee S, Pal JK (2009). Role of 5′- and 3′-untranslated regions of mRNAs in human diseases. Biol Cell.

[6] Farrugia DJ, Agarwal MK, Pankratz VS (2008). Functional assays for classification of BRCA2 variants of uncertain significance. Cancer Res.

[7] Walker LC, Whiley PJ, Couch FJ (2010). Detection of splicing aberrations caused by BRCA1 and BRCA2 sequence variants encoding missense substitutions: implications for prediction of pathogenicity. Hum Mutat.

[8] Cartegni L, Chew SL, Krainer AR (2002). Listening to silence and understanding non-sense: exonic mutations that affect splicing. Nat Rev Genet.

[9] Spurdle AB, Couch FJ, Hogervorst FB, Radice P, Sinilnikova OM (2008). Prediction and assessment of splicing alterations: implications for clinical testing. Hum Mutat.

[10] Lu M, Conzen SD, Cole CN, Arrick BA (1996). Characterization of functional messenger RNA splice variants of BRCA1 expressed in nonmalignant and tumor-derived breast cells. Cancer Res.

[11] Wang H, Shao N, Ding QM, Cui J, Reddy ES, Rao VN (1997). BRCA1 proteins are transported to the nucleus in the absence of serum and splice variants BRCA1a, BRCA1b are tyrosine phosphoproteins that associate with E2F, cyclins and cyclin dependent kinases. Oncogene.

[12] Wilson CA, Payton MN, Elliott GS, Buaas FW, Cajulis EE, Grosshans D, Ramos L, Reese DM, Slamon DJ, Calzone FJ (1997). Differential subcellular localization, expression and biological toxicity of BRCA1 and the splice variant BRCA1-delta11b. Oncogene.

[13] Orban TI, Olah E (2001). Expression profiles of BRCA1 splice variants in asynchronous and in G1/S synchronized tumor cell lines. Biochem Biophys Res Commun.

[14] Orban TI, Olah E (2003). Emerging roles of BRCA1 alternative splicing. Mol Pathol.

[15] Maniccia AW, Lewis C, Begum N (2009). Mitochondrial localization, ELK-1 transcriptional regulation and growth inhibitory functions of BRCA1, BRCA1a, and BRCA1b proteins. J Cell Physiol.

[16] Colombo M, Blok MJ, Whiley P (2014). Comprehensive annotation of splice junctions supports pervasive alternative splicing at the BRCA1 locus: a report from the ENIGMA consortium. Hum Mol Genet.

[17] Chen M, Manley JL (2009). Mechanisms of alternative splicing regulation: insights from molecular and genomics approaches. Nat Rev Mol Cell Biol.

[18] Tesoriero AA, Wong EM, Jenkins MA, Hopper JL, Brown MA, Chenevix-Trench G, Spurdle AB, Southey MC, kConFab (2005). Molecular characterization and cancer risk associated with BRCA1 and BRCA2 splice site variants identified in multiple-case breast cancer families. Hum Mutat.

[19] Antoniou AC, Durocher F, Smith P, Simard J, Easton DF, INHERIT BRCAs program members (2006). BRCA1 and BRCA2 mutation predictions using the BOADICEA and BRCAPRO models and penetrance estimation in high-risk French-Canadian families. Breast Cancer Res.

[20] Antoniou AC, Cunningham AP, Peto J (2008). The BOADICEA model of genetic susceptibility to breast and ovarian cancers: updates and extensions. Br J Cancer.

[21] de Garibay GR, Acedo A, García-Casado Z (2014). Capillary electrophoresis analysis of conventional splicing assays: IARC analytical and clinical classification of 31BRCA2 genetic variants. Hum Mutat.

[22] Houdayer C, Caux-Moncoutier V, Krieger S (2012). Guidelines for analysis in molecular diagnosis derived from a set of 327 combined in silico/in vitro studies on BRCA1 and BRCA2 variants. Hum Mutat.

[23] Whiley PJ, de la Hoya M, Thomassen M (2014). Comparison of mRNA splicing assay protocols across multiple laboratories: recommendations for best practicein standardized clinical testing. Clin Chem.

[24] Montagna M, Santacatterina M, Torri A, Menin C, Zullato D, Chieco-Bianchi L, D’Andrea E (1999). Identification of a 3 kb Alu-mediated BRCA1 gene rearrangement in two breast/ovarian cancer families. Oncogene.

[25] Sevcik J, Falk M, Macurek L (2013). Expression of human BRCA1Delta17-19 alternative splicing variant with a truncated BRCT domain in MCF-7 cells results in impaired assembly of DNA repair complexes and aberrant DNA damage response. Cell Signal.

[26] Mazoyer S (2005). Genomic rearrangements in the BRCA1 and BRCA2 genes. Hum Mutat.

[27] Bonnet C, Krieger S, Vezain M (2008). Screening BRCA1 and BRCA2 unclassified variants for splicing mutations using reverse transcription PCR on patient RNA and an ex vivo assay based on a splicing reporter minigene. J Med Genet.

[28] Gutiérrez-Enríquez S, Coderch V, Masas M, Balmaña J, Diez O (2009). The variants BRCA1 IVS6-1G>A and BRCA2 IVS15+1G>A lead to aberrant splicing of the transcripts. Breast Cancer Res Treat.

[29] Perrin-Vidoz L, Sinilnikova OM, Stoppa-Lyonnet D, Lenoir GM, Mazoyer S (2002). The nonsense-mediated mRNA decay pathway triggers degradation of most BRCA1 mRNAs bearing premature termination codons. Hum Mol Genet.

